# Supplementary material for: Insights into the Evolution of Spermatogenesis-Related Ubiquitin–Proteasome System Genes in Abdominal Testicular Laurasiatherians
Source: Genes (Basel). 2021 Nov 10;12(11):1780. doi: 10.3390/genes12111780 (PMC8620446; doi:10.3390/genes12111780)
Supplement: Supplementary file 1 [file genes-12-01780-s001.zip › genes-1423277-supplementary.pdf]

Supplementary

Table S1. 25 spermatogenesis-associated UPS candidate geness

Class of enzyme	Name	Testis phenotype of mutants
E1	*UBA1/UBE1*	Undefined
	UBA7/UBE1L	Undefined
E2	*UBE2A/HR6A*	Fertile; showing normal spermatogenesis
	UBE2B/HR6B	Infertility; sperm head shape anomalies, flagellar anomalies; apoptosis of primary spermatocytes in the first wave
E3	*RNF4*	Infertility; depletion of germ cells; age-dependent testicular atrophy
	RNF8	Infertility; defects in removal of whole nucleosomes during spermatogenesis due to reduced histone ubiquitination in the testis
	RNF19A	Undefined
	RAD18/RAD18Sc	Subfertility; defective MSCI; defective DSB repair during meiosis
	ANAPC1	Undefined
	MARCH7	Undefined
	MARCH10	Undefined
	CUL4B	Infertility; a progressive loss in germ cells; oligoasthenospermia; aberrant acrosomes in spermatids and nuclear morphology
	HERC4	Subfertility; angulated sperm tail; reduced sperm motility
	ITCH	Subfertility; delayed spermatid development; age dependent injure in spermatogenesis with increased apoptosis,
	Mei4	Infertility; inability of spermatocytes lack of crossing over; failure of chromosome aggregation on the metaphase plate resulting in arrest in the spermatocyte stage and subsequent apoptosis
	RFP	Undefined
	TMF1/ARA160	Infertility; lack of motility; lack of acrosome in the spermatozoa
Proteasome	*PSMA8*	Infertility; stalled in the middle stage of meiosis I
	PSME4	Subfertility; hypospermatocytogenesis; defective nucleosomal removal in apoptotic spermatocytes and spermatids
DUB	*USP2/UBP-testis*	Subfertility; lower motility in nutrient-deprived conditions; abnormal aggregation in

	elongated spermatids
USP14	Spermatid differentiation
UCHL1	Impaired spermatogenesis; decreased sperm concentration and motility; decreased spermatogonial stem cell proliferation and increased number of premeiotic germ cells
UCHL3	Increased germ cell loss and testicular atrophy following cryptorchid injury of the testis
UCHL5	Undefined
CYLD	Infertility; attenuation of early wave of germ cell apoptosis; spermatid deficiency

Table S2. Information of genes sequences in this study

Classification	Species name	Accession number						
		UCHL3	*PSMA8*	*UCHL5*	*USP14*	*USP2*	*RNF4*	*MARCH10*
Cetartiodactyla	*Tursiops truncatus*	XM 019921090.2	XM_019930480.2	XM_004322016.3	XM_019930560.1	XM_033861919.1	XM_033856537.1	XM_033848264.1
	Orcinus orca	XM_033414046.1	XM_004273735.2	XM_004275841.3	XM_033435365.1	XM_004273294.2	XM_033419602.1	XM_033438951.1
	Delphinapterus leucas	XM_030762626.1	XM_022586873.2	XM_022579206.1	XM_030760684.1	XM_022560281.2	XM_022554761.2	XM_022589088.2
	Physeter catodon	XM_028497491.1	XM_007118278.2	XM_028488075.1	XM_028480320.1	XM_007124219.3	XM_007105074.3	XM_024130194.2
	Balaenoptera acutorostrata	XM_007186210.1	XM_007197578.1	XM_007191161.2	XM_007191580.1	XM_007196672.1	XM_007172274.1	XM_007175799.1
	Bos taurus	XM_010810844.3	XM_002697717.5	XM_005216762.3	NM_001075189.1	NM_001046263.1	XM_024993426.1	XM_004432626.2
	Ovis aries	XM_027973697.1	XM_004020462.4	XM_004013585.4	XM_027961079.1	XM_027979322.1	XM_027971300.1	XM_024980354.1
	Capra hircus	XM_018056500.1	XM_018039666.1	XM_005690458.3	XM_018039483.1	XM_005689552.3	XM_018049833.1	XM_027974358.1
	Sus scrofa	XM_021065167.1	XM_021096195.1	DQ917642.1	XM_021096161.1	XM_005667434.3	NM_001044528.2	XM_018065317.1
	Vicugna pacos	XM_031684463.1	XM_006205089.3	XM_006203637.3	XM_006205118.2	XM_006207726.3	XM_015246870.2	XM_005653890.3
	Camelus ferus	XM_032496863.1	XM_006187170.3	XM_006186114.3	BLAST	XM_006175287.3	XM_014558714.2	XM_006199494.3
Perissodactyla	*Ceratotherium simum*	BLAST	BLAST	BLAST	BLAST	BLAST	XM_014788831.1	XM_006177436.3
	Equus caballus	XM_005601275.3	XM_001495329.4	XM_023647533.1	XM_023647532.1	BLAST	XM_023638492.1	XM_023652261.1
Carnivora	*Neomonachus schauinslandi*	XM_021697825.1	XM_021686265.1	XM_021686633.1	XM_021686046.1	XM_021696279.1	XM_021677716.1	XM_021678372.1
	Odobenus rosmarus	XM_012568190.1	XM_004411329.2	XM_004411339.2	XM_004411207.2	XM_004412584.1	XM_004396209.2	XM_012564311.1
	Canis lupus familiaris	XM_038431178.1	XM_038672432.1	XM_038448388.1	XM_038672506.1	XM_038664646.1	XM_038442196.1	XM_022422809.2

	Mustela putorius furo	XM_004754395.2	XM_004742862.2	XM_004751496.2	XM_004742809.2	XM_004750053.2	XM_013045800.1	XM_004749230.2
	Ailuropoda melanoleuca	XM_011234332.3	XM_002912678.4	XM_002923389.4	XM_002922622.4	XM_034665971.1	XM_002916543.4	BLAST
	Felis catus	XM_019828102.1	XM_003995084.4	XM_011291085.3	XM_003995057.5	XM_019811415.2	XM_023253532.1	XM_019818067.2
Chiroptera	*Pteropus vampyrus*	XM_023536381.1	XM_011360403.1	XM_011360466.2	XM_023530834.1	XM_023525828.1	XM_011374699.2	XM_011368587.2
	Pteropus alecto	XM_025048193.1	XM_006920016.1	XM_006920041.3	XM_025038629.1	XM_025047333.1	XM_006919032.3	XM_006912330.2
	Myotis davidii	XM_015559224.1	XM_006764885.2	XM_006761341.2	XM_006761874.2	XM_006758700.2	XM_006760124.2	XM_006770277.2
	Myotis lucifugus	XM_023751722.1	XM_006104470.1	XM_006103195.3	XM_006097384.3	NA	XM_023745402.1	XM_014458702.2
	Eptesicus fuscus	XM_028149045.1	XM_008148334.2	XM_008149834.2	XM_008160165.2	XM_008150072.2	XM_008150907.2	XM_028157441.1
Eulippotyphla	*Condylura cristata*	XM_012723373.1	XM_004683989.1	XM_004685461.2	XM_004683774.2	XM_004689174.1	XM_012724164.1	XM_004693338.2
	Erinaceus europaeus	XM_007531947.1	BLAST	XM_007539744.2	XM_007537081.2	XM_007523576.2	XM_016194572.1	XM_016190226.1

Classification	Species name	Accession number						
		ANAPC1	*CUL4B*	*CYLD*	*HERC4*	*ITCH*	*Mei4*	*PSME4*
Cetartiodactyla	*Tursiops truncatus*	XM_033838554.1	XM_033849375.1	XM_019932498.2	XM_019936183.2	XM_019950661.2	XM_033836630.1	XM_019942853.2
	Orcinus orca	XM_033399994.1	XM_033416233.1	XM_004264892.3	XM_033407638.1	XM_033433515.1	XM_033428723.1	XM_004286518.3
	Delphinapterus leucas	XM_022599309.2	XM_022561852.1	XM_030761535.1	XM_022590698.2	XM_022592619.2	XM_022590481.1	XM_022597155.1
	Physeter catodon	XM_028496614.1	XM_007122043.3	XM_028477431.1	XM_024132087.2	XM_028498438.1	XM_024132967.1	XM_024118664.2
	Balaenoptera acutorostrata	XM_007197378.2	XM_007178609.1	XM_028162892.1	XM_007167806.2	XM_007193258.1	XM_007167979.1	XM_007190021.1
	Bos taurus	XM_005212369.4	XM_002699510.5	NM_001046417.1	NM_001076894.3	BC133503.1	XM_024996921.1	NM_001205952.1
	Ovis aries	XM_027967124.1	XM_015105033.2	XM_015100436.2	XM_015104378.2	XM_027977101.1	XM_027972295.1	XM_015094386.2
	Capra hircus	XM_018054804.1	XM_018044199.1	XM_013971026.2	XM_018042293.1	XM_005688476.3	XM_018053506.1	XM_005686603.3

	Sus scrofa	XM_021087035.1	XM_013986358.2	XM_021094193.1	XM_021072872.1	GACC01000520.1	XM_021092258.1	XM_003125150.6
	Vicugna pacos	XM_031692069.1	XM_006215899.3	XM_031680579.1	XM_006212920.3	XM_031687981.1	XM_031679874.1	XM_006211270.3
	Camelus ferus	XM_014558298.2	XM_032476122.1	XM_032486293.1	XM_032491503.1	XM_006190776.3	XM_032484497.1	XM_006174442.3
Perissodactyla	*Ceratotherium simum*	XM_014791619.1	BLAST	XM_014788384.1	XM_014784913.1	XM_014787535.1	XM_014781074.1	BLAST
	Equus caballus	XM_023618721.1	XM_023634079.1	XM_023636965.1	XM_001503586.4	XM_001916721.5	XM_023650676.1	XM_023619106.1
Carnivora	*Neomonachus schauinslandi*	XM_021697340.1	XM_021686083.1	XM_021705825.1	XM_021700187.1	XM_021689031.1	XM_021693619.1	XM_021701246.1
	Odobenus rosmarus	XM_012562159.1	XM_004406921.2	XM_004408513.2	XM_004393708.2	XM_012564988.1	XM_012562208.1	XM_004403665.2
	Canis lupus familiaris	XM_532958.7	XM_038450783.1	XM_005617568.4	XM_038662710.1	XM_022409130.2	XM_014118115.3	XM_038680568.1
	Mustela putorius furo	XM_004770988.2	XM_004762060.2	XM_004744171.2	XM_004777827.2	XM_013053385.1	XM_004752830.2	XM_004775684.2
	Ailuropoda melanoleuca	XM_011231155.3	XM_011229099.3	XM_011218427.3	XM_034662593.1	XM_019796585.2	XM_034648277.1	XM_034659133.1
	Felis catus	XM_003984262.5	XM_011279431.3	XM_023244894.1	XM_011287365.2	XM_003983597.5	XM_006931862.2	XM_023251673.1
Chiroptera	*Pteropus vampyrus*	XM_023521936.1	XM_011378241.2	XM_023530669.1	XM_023520115.1	XM_011378287.2	XM_011364921.2	XM_011363964.2
	Pteropus alecto	XM_006909446.3	XM_015593967.2	XM_025044597.1	XM_025049386.1	XM_006921736.3	XM_006926811.2	XM_006909599.2
	Myotis davidii	XM_006769219.2	XM_006767808.2	XM_015572642.1	XM_015562117.1	XM_006764581.2	XM_006772205.2	XM_015557867.1
	Myotis lucifugus	XM_023764404.1	XM_006082148.3	XM_006105966.3	XM_023754626.1	XM_006089425.2	XM_023755201.1	XM_023746830.1
	Eptesicus fuscus	XM_008159952.2	XM_028132046.1	XM_028126899.1	XM_028151457.1	XM_008149714.2	XM_008139546.2	XM_028139915.1
Eulippotyphla	*Condylura cristata*	XM_012727535.1	XM_012727233.1	XM_004693563.2	XM_004680846.2	XM_004687268.2	XM_012730908.1	XM_004686198.2
	Erinaceus europaeus	XM_007532163.2	XM_016191402.1	XM_016194777.1	XM_007524167.2	XM_016187962.1	XM_007521946.2	XM_007527952.2

Accession number

Classification	Species name	*RAD18*	*RFP*	*UCHL1*	*RNF19A*	*TMF*	*UBA1*	*UBA7*
Cetartiodactyla	*Tursiops truncatus*	XM_033865323.1	XM_004330846.3	XM_019928270.2	XM_019925060.2	XM_004330307.3	XM_033850069.1	XM_033864980.1
	Orcinus orca	XM_033437223.1	XM_004286088.2	XM_004268322.2	XM_033438195.1	XM_033424796.1	XM_004281995.2	XM_004267814.3
	Delphinapterus leucas	XM_022564695.1	XM_022562132.2	XM_022565351.2	XM_022584691.2	XM_022572588.2	XM_022556334.2	XM_022573094.1
	Physeter catodon	XM_024124120.2	XM_007110733.3	XM_024117960.2	XM_007104846.3	XM_007105914.3	XM_007104174.3	XM_007113963.3
	Balaenoptera acutorostrata	XM_007192496.1	XM_007179083.2	XM_007178766.1	XM_028164300.1	XM_007192426.1	XM_007183644.2	XM_007168713.1
	Bos taurus	XM_005222442.4	NM_001075799.1	XM_005207872.4	NM_001191331.1	NM_001206260.1	NM_001102477.1	NM_001012284.1
	Ovis aries	XM_004018298.3	XM_004019024.4	XM_004009789.3	XM_027973176.1	XM_004018337.4	XM_004022137.3	XM_027957618.1
	Capra hircus	XM_005695644.2	XM_018038518.1	XM_005681551.3	XM_018058303.1	XM_005695752.3	XM_018043651.1	XM_005695943.2
	Sus scrofa	NM_001142833.1	XM_003128235.5	AY459532.2	XM_013996574.2	XM_021069200.1	XM_013990866.2	XM_003132214.4
	Vicugna pacos	XM_006206888.3	XM_006215241.2	XM_015244020.2	XM_031691200.1	XM_006196477.3	XM_006213407.3	XM_006196316.3
	Camelus ferus	XM_032458862.1	XM_006192028.3	XM_006191755.3	XM_032468187.1	XM_032458108.1	XM_032474489.1	XM_006174109.3
Perissodactyla	*Ceratotherium simum*	XM_004442198.2	XM_004441740.2	BLAST	XM_014788090.1	BLAST	BLAST	BLAST
	Equus caballus	XM_023620038.1	XM_023624125.1	NM_001081820.1	XM_023648770.1	XM_014731465.2	XM_023634144.1	XM_005600650.3
Carnivora	*Neomonachus schauinslandi*	XM_021695067.1	XM_021697099.1	XM_021697826.1	XM_021688697.1	XM_021694908.1	XM_021679602.1	XM_021686973.1
	Odobenus rosmarus	XM_004392719.1	XM_004411293.1	XM_004396136.2	XM_004402351.2	XM_012563807.1	XM_004396744.1	XM_004399254.1
	Canis lupus familiaris	XM_038565773.1	XM_038446650.1	XM_038480474.1	XM_022426584.2	XM_038427176.1	XM_038449878.1	XM_038427722.1
	Mustela putorius furo	XM_004738431.2	XM_004779969.2	XM_004764057.2	XM_013064759.1	XM_004738330.2	XM_004754955.2	XM_004760621.2
	Ailuropoda melanoleuca	XM_034658694.1	XM_002928641.3	XM_002926504.4	XM_002913982.4	XM_002924401.4	XM_002917778.4	XM_002920564.4

	Felis catus	XM_003982447.4	XM_023254671.1	XM_011281913.2	XM_006943325.3	XM_003982410.5	XM_004000424.5	XM_006928773.4
Chiroptera	*Pteropus vampyrus*	XM_011370651.2	XM_011381952.2	XM_011362779.1	XM_011376181.2	XM_011358479.2	XM_011380062.2	XM_011364732.2
	Pteropus alecto	XM_006917575.3	XM_025044337.1	XM_015597498.1	XM_006916648.3	XM_015593757.2	XM_006906892.3	XM_015587154.2
	Myotis davidii	XM_015560350.1	BLAST	XM_006759327.2	XM_006760292.2	XM_015567947.1	XM_015561992.1	BLAST
	Myotis lucifugus	XM_023749718.1	BLAST	XM_006088473.3	XM_023763614.1	XM_023758102.1	XM_023760927.1	XM_014448405.2
	Eptesicus fuscus	XM_008151717.2	BLAST	XM_008140249.2	XM_008148503.2	XM_008154157.2	XM_028127656.1	XM_008157503.2
Eulippotyphla	*Condylura cristata*	XM_012732091.1	XM_004695445.2	XM_004681331.2	XM_004679693.2	XM_004675990.2	XM_004689974.2	XM_004676152.2
	Erinaceus europaeus	XM_007522261.2	XM_007538640.2	XM_007526999.2	XM_007532786.2	XM_007523323.1	XM_007529642.2	XM_016190081.1

Classification	Species name	Accession number			
		UBE2A	*UBE2B*	*RNF8*	*MARCH7*
Cetartiodactyla	*Tursiops truncatus*	XM_004329769.3	XM_004326503.2	XM_019949791.2	XM_019922517.2
	Orcinus orca	XM_004275810.3	XM_004282119.3	XM_004267655.2	XM_033421819.1
	Delphinapterus leucas	XM_022562959.2	XM_022588824.1	XM_022581794.1	XM_022569836.2
	Physeter catodon	XM_024128099.2	XM_007107071.3	XM_024122061.2	XM_024122319.2
	Balaenoptera acutorostrata	BLAST	XM_007172150.2	XM_007197935.2	XM_007183151.2
	Bos taurus	BC102100.1	NM_001037459.2	NM_001046216.1	XM_015475216.2
	Ovis aries	BLAST	XM_004008801.3	XM_027958350.1	XM_027964909.1
	Capra hircus	XM_005700284.3	XM_005682942.3	XM_018039174.1	XM_013969446.2
	Sus scrofa	XM_001927284.4	NM_001257356.1	XM_005665930.3	XM_021074770.1
	Vicugna pacos	XM_015243190.2	XM_006212804.3	XM_031688483.1	XM_031678470.1

	Camelus ferus	XM_032476127.1	XM_006179617.3	XM_032462982.1	XM_032479549.1
Perissodactyla	*Ceratotherium simum*	BLAST	BLAST	XM_004424191.2	XM_014785916.1
	Equus caballus	XM_001492266.6	XM_001504396.4	XM_023624866.1	XM_023622588.1
Carnivora	*Neomonachus schauinslandi*	XM_021686228.1	XM_021702227.1	XM_021684698.1	XM_021703029.1
	Odobenus rosmarus	XM_004405225.2	XM_004405451.2	XM_004409774.2	XM_004394827.2
	Canis lupus familiaris	XM_038450727.1	XM_038616048.1	XM_038683373.1	BLAST
	Mustela putorius furo	XM_004762093.2	XM_004744962.2	BLAST	XM_004743915.2
	Ailuropoda melanoleuca	XM_034649995.1	XM_002912915.4	XM_011232114.3	BLAST
	Felis catus	XM_004000827.5	XM_023255298.1	XM_023253930.1	XM_003990797.5
Chiroptera	*Pteropus vampyrus*	XM_011380198.2	XM_011362314.2	XM_023527546.1	XM_011358949.2
	Pteropus alecto	XM_006904701.2	XM_006923125.3	XM_006907307.3	XM_015596530.2
	Myotis davidii	XM_006762329.2	XM_006753550.2	XM_015571884.1	XM_006766473.2
	Myotis lucifugus	XM_006082177.3	XM_006086593.3	XM_006104390.3	XM_006083224.3
	Eptesicus fuscus	XM_008156842.2	XM_008142249.2	XM_028161192.1	XM_028141141.1
Eulippotyphla	*Condylura cristata*	XM_004685745.2	XM_004686729.2	XM_012723334.1	XM_012728344.1
	Erinaceus europaeus	XM_007537210.2	XM_007519906.2	XM_016185883.1	XM_007537376.2
Primates	*Homo sapiens*			NM_003958.4	NM_001376235.1
Rodentia	*Rattus norvegicus*			NM_001025727.1	XM_039104832.1
Lagomorpha	*Oryctolagus cuniculus*			XM_008262860.2	XM_008258623.2

Scandentia	*Tupaia chinensis*	XM_027771887.1	XM_006160292.3
Ameridelphia	*Monodelphis domestica*	XM_003340356.3	XM_007494240.1
Proboscidea	*Loxodonta africana*	XM_003340356.3	XR_775560.2
Sirenia	*Trichechus manatus*	XM_023734147.1	XM_023730436.1
Tubulidentata	*Orycteropus afer*	XM_007939460.1	XM_007941827.1
Cingulata	*Dasypus novemcinctus*	XM_004484074.3	XM_004463900.2
Monotremata	*Ornithorhynchus anatinus*	XM_029047718.2	XM_029071808.2

BLAST: obtained from the local database blast; NA: Not available

Table S3. Positive selection analyses of spermatogenesis-related UPS genes by free-ratio model

Gene	Branch	dN	dS	ω value
UCHL3	*P.catodon*	0.020879	0.006814	3.06434
PSMA8	LCA of *T.truncatus* & *O.orca*	0.007289	0.005223	1.39569
	LCA of *O.orca* & *D.leucas*	0.007280	0.005029	1.44762
USP14	*O.orca*	0.007106	0.006319	1.1245
MARCH7	LCA of *E.europaeus* & *C.cristata*	0.003624	0.001498	2.41938
USP2	LCA of *E.europaeus* & *C.cristata*	0.006208	0.000793	7.82879
RNF4	*C.simum*	0.009778	0.009055	1.07985
MARCH10	*C.ferus*	0.012444	0.010437	1.19232
	LCA of *M.putorius* & *N.schauinslandi*	0.002919	0.001446	2.01887
	LCA of *D.leucas* & *O.orca*	0.013495	0.005334	2.53004
	LCA of *P.vampyrus* & *M.davidii*	0.012336	0.007176	1.71906
UBA7	*O.rosmarus*	0.018217	0.014629	1.24524
	LCA of *F.catus* & *C.simum*	0.006639	0.000891	7.44929
RNF8	LCA of *T.truncatus* & *O.orca*	0.002884	0.002358	1.22295
	LCA of *F.catus* & *M.davidii*	0.012038	0.008742	1.37713
RAD18	LCA of *E.europaeus* & *C.cristata*	0.000408	0.000211	1.93453
	M.lucifugus	0.007201	0.002231	3.22728
PSME4	LCA of *F.catus* & *M.davidii*	0.001314	0.000872	1.50661
Mei4	*P.catodon*	0.013724	0.007622	1.80062
	LCA of *F.catus* & *A.melanoleuca*	0.014740	0.005909	2.49452
ITCH	LCA of *F.catus* & *M.davidii*	0.000491	0.000418	1.17442
ANAPC1	LCA of *T.truncatus* & *O.orca*	0.000241	0.000026	9.35368
	LCA of *F.catus* & *M.davidii*	0.001235	0.000593	2.08056
TMF	*C.ferus*	0.005002	0.004523	1.10605
	Capra hircus	0.000406	0.000255	1.59098

Table S4. Positive selection analyses of spermatogenesis-related UPS genes by two-ratio model

Gene	Branch	ω value	2ΔlnL	*p* value *p*<0.05	Adjusted *p*
UCHL3	*P.catodon*	4.00226	30.859968	2.77334E-08	6.93335E-07
	LCA of *B.acutorostrata* & *O.orca*	1.18056	6.36654	0.011629212	0.096910104
PSMA8	LCA of *O.orca* & *T.truncatus*	1.42432	11.14066	0.000844558	0.021113959
	LCA of *O.orca* & *D.leucas*	1.556	11.159808	0.000835886	0.010448572
USP14	*O.orca*	1.09446	19.426548	1.04544E-05	6.93335E-07
MARCH7	LCA of *E.europaeus* & *C.cristata*	6.68952	4.00992	0.045233297	0.161547489
MARCH10	LCA of *D.leucas* & *O.orca*	2.6436	10.459168	0.001220422	0.010170186
	C.ferus	1.29721	4.98311	0.02559593	0.127979651
UBA7	*O.rosmarus*	1.30561	15.875692	6.76419E-05	0.001691049
	LCA of *F.catus* & *C.simum*	8.03186	5.699768	0.016967156	0.424178889
Mei4	*P.catodon*	1.7988	5.108186	0.023813206	0.198443383
	LCA of *C.lupus* & *A.melanoleuca*	2.75833	7.087416	0.007762709	0.097033868
	LCA of *F.catus* & *M.davidii*	3.71722	5.206544	0.022502021	0.112510105

Table S5. Rapid evolution analyses of spermatogenesis-related UPS genes by two-ratio model

Gene	2ΔlnL	ω in scrotal testicular mammals	ω in abdominal testicular mammals	*p* value *p*<0.05	Adjusted *p*
UBE2A	11.655552	0.02689	0.11116	0.000640112	0.008001401
UCHL3	6.81078	0.16734	0.07602	0.009060918	0.045304589
HERC4	8.487604	0.0506	0.08967	0.003575744	0.029797867
PSME4	8.297916	0.0517	0.07572	0.00396906	0.024806624
PSMA8	4.006612	0.05689	0.09543	0.045322138	0.161864779
USP2	4.04734	0.09427	0.1306	0.044241004	0.184337517
RFP	24.878692	0.03784	0.00731	6.10535E-07	1.52634E-05

Table S6. Positive selection analyses of spermatogenesis-related UPS genes by branch-site model

Gene	Branch	2ΔlnL	*p* value *p*<0.05	Adjusted *p*	ω value	Positively selected sites (PP > 0.8)
UCHL3	*P.catodon*	4.27495	0.038678102	0.966952545	6.1502	5 R 0.979* 8 P 0.980* 31 F 0.983* 224 P 0.982* 236 A 0.982* 252 P 0.981* 270 I 0.982* 273 C 0.980* 278 E 0.982* 284 L 0.979*
PSMA8	LCA of *T.truncatus* & *O.orca*	4.249994	0.039250469	0.981261729	5.33697	19 L 0.946 40 A 0.938 214 G 0.948
MARCH10	LCA of *D.leucas* & *O.orca*	4.236318	0.039567891	0.329732423	11.68875	75 K 0.936 82 G 0.890 106 H 0.933 119 N 0.937 147 I 0.922 221 K 0.935 551 H 0.922 608 N 0.919 691 S 0.933 734 A 0.935 776 G 0.919
RAD18	*C.cristata*	6.042904	0.013962299	0.174528736	4.16586	6 E 0.949 110 K 0.955* 174 S 0.951* 501 N 0.913
CYLD	*M.davidii*	4.511314	0.033671364	0.42089205	8.56895	111 S 0.979* 309 Q 0.960*

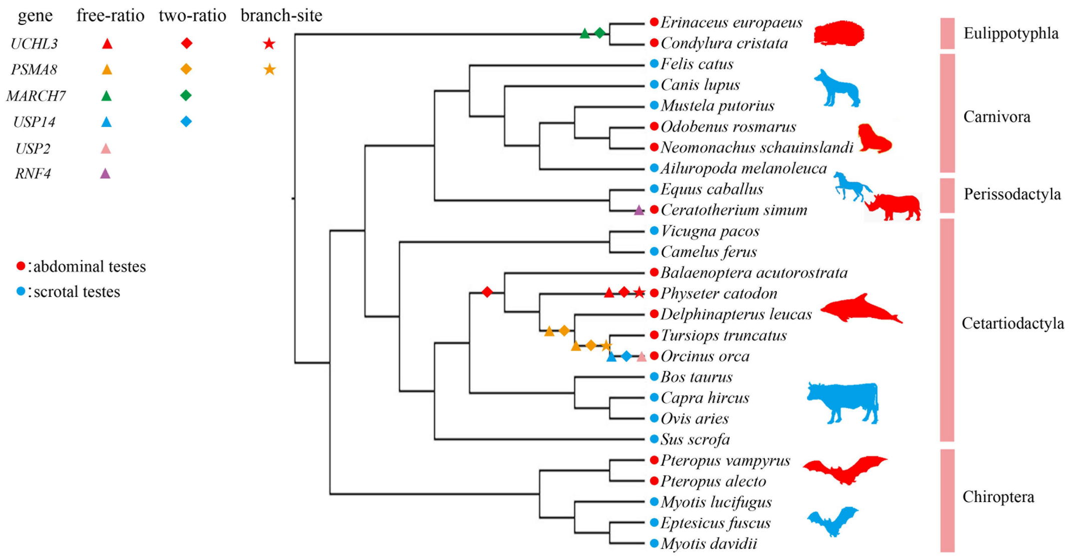

Figure S1. All abdominal testicular branches-specific positively selected genes. Positively selected branches identified by the free-ratio model and two-ratio model are represented by triangles and diamonds, respectively. The six abdominal testicular branches-specific positively selected genes are marked with different colors: *UCHL3* (red), *PSMA8* (orange), *USP14* (blue), *MARCH7* (green), *USP2* (pink) and *RNF4* (purple). The mammals with abdominal testes are shown in red, and mammals with scrotal testicular are in blue. *P* value tested by LRT<0.05.

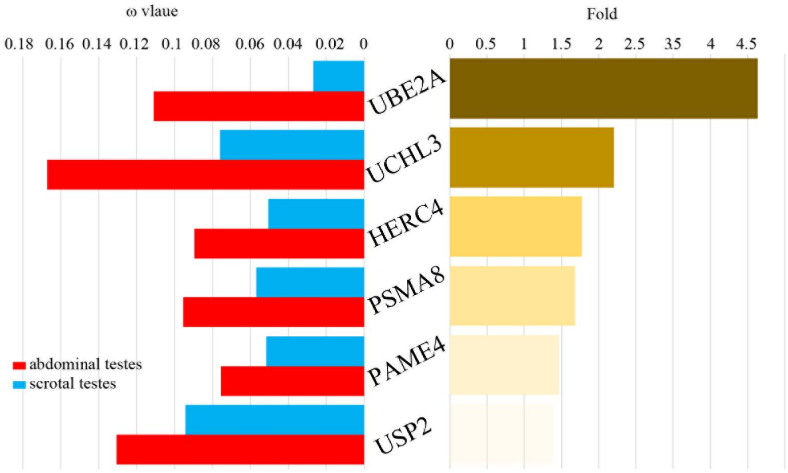

Figure S2. All rapidly evolving genes in abdominal testicular lineages. The nested branch model (two-ratio model) was used to calculate the selection pressure of the foreground branch (abdominal testicular species) and the foreground branches (scrotal testicular species). The left side showes the ω values of the four accelerated genes for the abdominal testicular and the scrotal testicular species. The ω values of all the genes for the abdominal testicular species

are greater than those of the scrotal testicular species. The right side shows the difference between the ω values of the abdominal and the scrotal testicular species of the four genes, sorted according to the difference from high to low. *P* value tested by LRT<0.05.

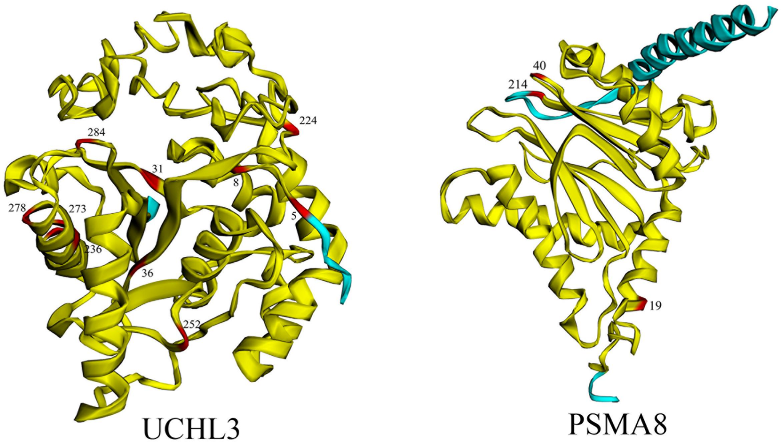

Figure S3. Distribution of positively selected sites on the 3D structure of proteins. The yellow represents the domain sequence, the red represents the positive selection site, and the blue represents the non-domain sequence. *P* value tested by LRT<0.05. Posterior probabilities ≥ 0.8.
